# Fast 3D movement of a laser focusing spot behind scattering media by utilizing optical memory effect and optical conjugate planes

**DOI:** 10.1038/s41598-019-56214-3

**Published:** 2019-12-20

**Authors:** Vinh Tran, Sujit K. Sahoo, Cuong Dang

**Affiliations:** 1 Centre for Optoelectronics and Biophotonics (COEB), School of Electrical and Electronic Engineering, The Photonics Institute (TPI), Nanyang Technological University Singapore, 50 Nanyang Avenue, Singapore, 639798 Singapore; 2 School of Electrical Science, Indian Institute of Technology Goa, At Goa College Engineering Campus, Farmagudi, Ponda, Goa 403401 India

**Keywords:** Optical imaging, Imaging and sensing

## Abstract

Controlling light propagation intentionally through turbid media such as ground glass or biological tissue has been demonstrated for many useful applications. Due to random scattering effect, one of the important goals is to draw a desired shape behind turbid media with a swift and precise method. Feedback wavefront shaping method which is known as a very effective approach to focus the light, is restricted by slow optimization process for obtaining multiple spots. Here we propose a technique to implement feedback wavefront shaping with optical memory effect and optical 4f system to speedy move focus spot and form shapes in 3D space behind scattering media. Starting with only one optimization process to achieve a focusing spot, the advantages of the optical configuration and full digital control allow us to move the focus spot with high quality at the speed of SLM frame rate. Multiple focusing spots can be achieved simultaneously by combining multiple phase patterns on a single SLM. By inheriting the phase patterns in the initial focusing process, we can enhance the intensity of the focusing spot at the edge of memory effect in with 50% reduction in optimization time. With a new focusing spot, we have two partially overlapped memory effect regions, expanding our 3D scanning range. With fast wavefront shaping devices, our proposed technique could potentially find appealing applications with biological tissues.

## Introduction

Delivering high power light into a small region inside biological tissues or translucent materials can allow us to do optical imaging, laser cutting, or light therapy. One can simply utilize infrared light (optical window for biological tissues) which has less absorption and scattering effect to focus deeper inside the body^1,2^. However, the most interesting light matter interactions happen at the spectrum region where the scattering effect is inevitable. Such strong scattering effect distorts the directionality, phase and intensity of transmitted light field; challenges our efforts to focus light through scattering media. A solution to control light to overcome scattering effects in various media such as skin, biological tissue or frosted glass could provide a useful approach to enable many photonic applications in various fields such as biomedical imaging or advanced manufacture.

Wavefront shaping technique is one of the most common and efficient approaches to solve the random scattering effect. The wavefront of incoming laser beam is shaped by spatial light modulators (SLM) or digital micro-mirror devices (DMD)^3,4^ to cancel out the scattering effect. Multiple strategies for finding the wavefront to shape the incoming beam and achieve a tight focusing spot have been demonstrated. Measuring the transmission matrix (TM) to map inputs with outputs allows us to calculate the correct wavefront input and produce a desired light pattern behind the turbid medium^4–10^. Q. Zhao *et al*.^11^ combines TM with PSF modulation^12^ and computer-generated holography (CGH)^13^ to generate focus spots in 3D space behind scattering media. Utilizing time reversal property of light, one can measure the scattered light field then create its phase conjugated light field at the same position to travel backward through the scattering medium and generate an original pattern^14–21^. This digital optical phase conjugation (DOPC) method has been proven with not only phase or amplitude modulation but also polarization modulation^22^ with equivalent results at much lower cost. Recently, angular-spectrum model has been demonstrated to trace the field propagation to investigate the focusing process in DOPC^23^. These methods require measuring the scattered light phase with a complex and sensitive optical setup. In addition, the digital optical phase conjugation method requires a critical alignment for pixel-by-pixel-matching between the phase measurement device and the phase modulation device. Another powerful and simple approach to create a spot behind scattering media is an optimization technique, which considers the scattering medium as a black box, thus, no strict requirement for optical alignment^3,21,24,25^. With the help of a guiding star on the other side of scattering media, we can build a feedback loop with genetic algorithm (GA) to shape the incident wavefront and achieve a light focusing spot.

Unlike the TM approach, the phase conjugation method or the optimization approach is limited to a single focus point/pattern for each measured or optimized input, respectively. A solution for moving the focus spot around or drawing a pattern with the focus spot requires multiple measurements for the phase conjugation approach^14–16,19–21^ or multiple optimization processes^26^, which would be time consuming. Starting with only a single optimized phase pattern for generating an initial focus spot, our previous approach utilizing the optical memory effect^27–29^ could focus a portion of a laser beam through a scattering medium into any other spots on the 2D plane, which is perpendicular to the optical axis. By tilting and shifting the pre-optimized phase pattern digitally, we can aim to any spots within the memory effect region^30^. However the further the target spot is from the initial spot, the smaller the laser portion is utilized. This limited utilization of laser beam together with the small memory effect region make the intensity of focusing spot reduced quickly with the distance from the target spot to the initial spot. Here, we demonstrate a method that utilize a 4F optic system to swiftly move the focus point in not only the 2D plane but also the 3D memory effect volume with full utilization of the laser beam. We are able to create any desired 3D contours on the other side of the scattering medium by moving the focus spot sequentially or generating multiple spots simultaneously. More importantly, the low intensity of the focus point at the edge of memory effect region can be quickly optimized with GA by inheriting the SLM phase patterns from the original optimization. Fast optimization process for the second spot enlarges the region beyond the memory effect limitation for our moving spot. The simplicity and advantages of our method can suggest a practical approach for many applications such as opto-genetic excitation, minimal invasive laser surgery, or imaging.

## Experiment Setup

**Figure 1:**
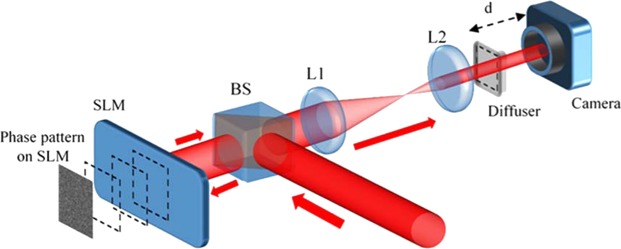
Experimental setup to control light through scattering media. BS: Non-polarized beam splitter. L1, L2: Convex lenses with focal length f_1_ = 150 mm and f_2_ = 50 mm, respectively. d: distance between diffuser and camera.

The experiment setup with 4F system is illustrated in Fig. 1. An expanded He-Ne laser beam with wavelength of 632.8 nm is guided by a non-polarized beam splitter to shine at the center of a SLM. The laser beam after reflecting on SLM, passes through an optical system of two convex lenses, L1 and L2, with focal lengths of 150 mm and 50 mm, respectively. A ground glass diffuser (Thorlabs, N-BK7), used as a turbid medium, is placed at the focal plane of L2 while the SLM is at the focal plane of L1 to create a 4F system where the SLM plane is relayed on the diffuser plane. The SLM’s plane and diffuser’s plane are conjugated with magnification of 1/3 to increase the spatial sampling rate of the SLM on the diffuser effectively. A monochromic CMOS camera (Thorlabs DCC1645C) is set behind the diffuser at an observation plane to provide feedback signals for optimization process and then observe the quality of the focus spots. Moving the camera along the optical axis allows us to visualize the focus spots in 3D.

## Result and Discussion

### Initial focus spot by genetic algorithm

For creating an initial focus spot behind a scattering medium, we utilize the feedback wavefront shaping technique with genetic algorithm (GA)^3^. In our setup, we combine 2 by 2 SLM pixels to be one controllable element for shaping the wavefront, so there are 540 × 540 elements. The typical GA iteratively shapes the wavefront of input laser beam to increase the brightness contrast between the desired position and average background, which is defined as peak-to-background ratio (PBR)^31^ as below:

1 $$PBR={I}_{S}/\overline{{I}_{B}}$$

where *I*_*S*_ is intensity of the focus point; $\overline{{I}_{B}}$ is the mean value of background intensity. With our uncooled 8-bit CMOS camera, the measurement fluctuation of pixel intensity during the optimization could largely affect the results. Here, we take an average intensity of 5 by 5 pixels around the target focus spot as *I*_*S*_ and $\overline{{I}_{B}}$ is the mean value of the captured image excluding 25 pixels counted for *I*_*S*_. The area of 5 by 5 pixels is chosen to cover the diffraction limit area of the focus spot and the averaging strategy can minimize the effect of random noise in intensity measurement at pixel level. This make our GA more stable and converged faster.

**Figure 2:**
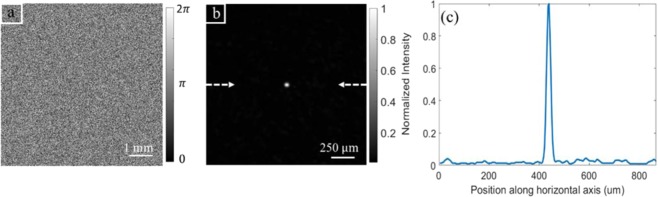
Shaping the incident beam wavefront to create a focus spot behind a scattering medium by GA. (**a**) Optimized phase pattern on SLM. (**b**) The optimized focus spot, the two arrows show the cross section line to measure the intensity. (**c**) Intensity profile along the horizontal line through the spot, presented by the two arrows in (**b**).

The result of feedback wavefront shaping with GA for creating a focus spot behind scattering medium is illustrated in Fig. 2. Our GA achieve the focus spot size (FWHM) of 20 μm with the brightest pixel at the center of 5 by 5 pixel area. The ratio between intensity of the brightest focus pixel and average background intensity is 112, which reaches the limitation of our 8-bit camera because the detected intensity on each pixel is already from 1 to 5 counts in a dark condition (closing the camera cap). A better camera with higher bit depth, higher photon capacity and lower noise (cooled sensor chip) would allow us to get better results. The quality of focus spot can also be improved by utilizing more elements for optimization and extending the number of iteration loops. However, this will cost more optimization time. With our current parameters, the optimizing time is about 10 minutes; running repeated optimization process is not feasible for creating multiple spots in 3D space or drawing desired shapes. We would like to note that the speed of our optimization process is largely limited by SLM updating rate and the readout time of our camera.

### Moving focus spot inside memory effect region

Here, we utilize optical memory effect to move the focus spot to multiple positions swiftly with only one initial optimization process. The position of the initial focus spot is considered at the origin of a Cartesian coordinate. In order to move the focus spot to a position (x, y) on the camera, incoming wavefront on diffuser’s surface needs to be tilted around X-axis and Y-axis by an angle α_x_ and α_y_, respectively: $\tan ({{\rm{\alpha }}}_{{\rm{x}}})={\rm{x}}/{\rm{d}}$ and $\tan ({{\rm{\alpha }}}_{{\rm{y}}})=\,y/{\rm{d}},$ where d is the distance between the camera plane and the diffuser. Because camera surface is sampled by a 2D pixel array, then the position x and y can be expressed by: x = n_x_ × p and y = n_y_ × p where n_x_ and n_y_ are number of pixels moved in X-axis and Y-axis, p is the pixel size of camera. The magnification from the SLM plane to the diffuser plane is 1/3, hence the real angles for tilting phase pattern on SLM are β_x_ = α_x_/3 and β_y_ = α_y_/3. Precise measurement of the distance d directly is challenging. Here, by a quick scanning process with several tilting angles β_x_ and β_y_ and observing the spot positions (x and y) on camera, the distance d can be estimated precisely to be 3.91 cm in our experimental setup. From here, the phase profile on SLM for moving the focus spot in 2D plane can be calculated easily as the combination of original optimized phase pattern and a tilting phase pattern.

**Figure 3:**
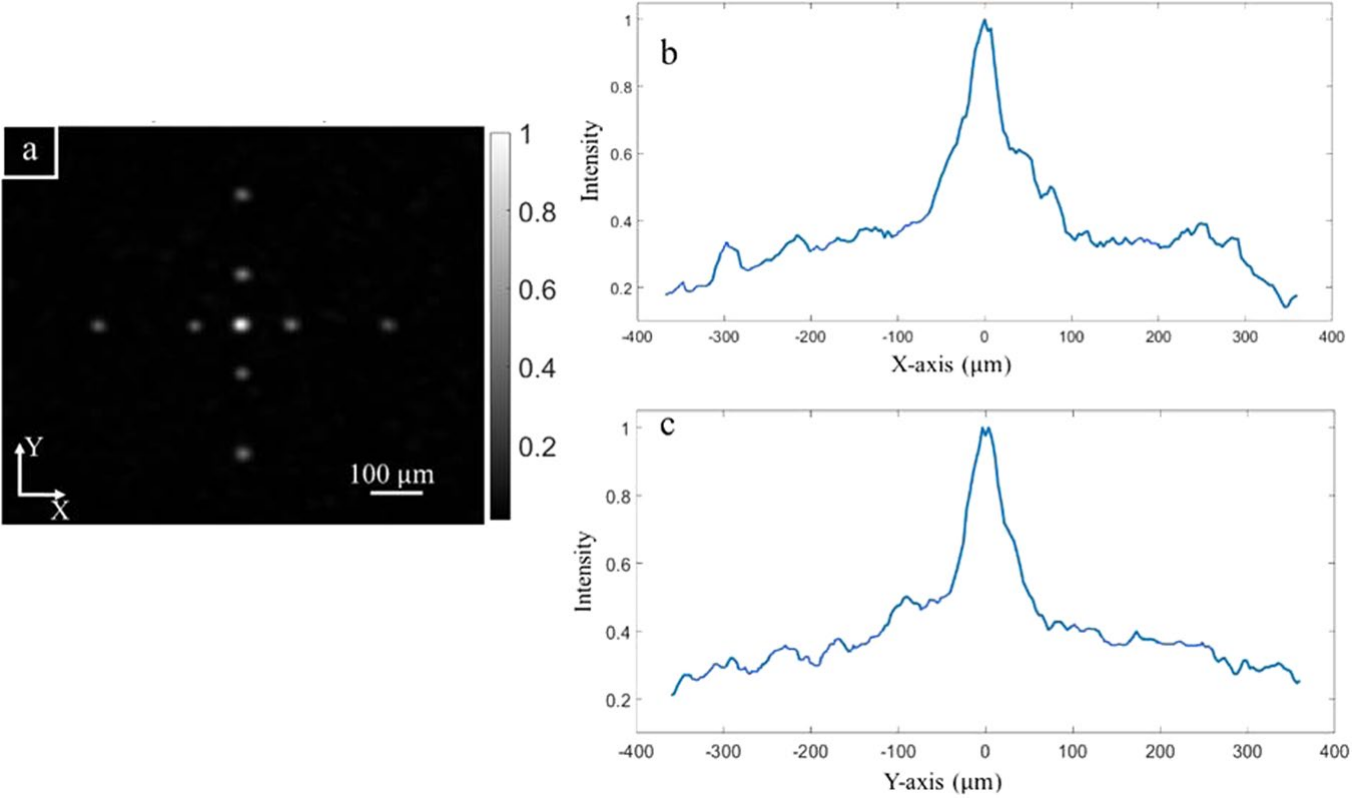
Intensity distribution of moving spot in a 2D plane. (**a**) Image of focus spots in different positions. Relative intensity of the moving spot along X-axis (**b**) and Y-axis (**c**).

Moving the focus spot based on memory effect is presented in Fig. 3 with the distribution of intensity in X-Y plane. The brightest spot at the center of the image (Fig. 3a) is the original focusing spot, which is optimized by GA. All other dimmer spots are achieved by adding the appropriate tilting phase on the original phase pattern. We achieve one spot for each calculated SLM pattern then multiple result images are superposed to present as a single image in Fig. 2a. The relative intensity of various spot along X and Y direction compared to the original spot is presented in Fig. 3b,c, respectively. The intensity is quickly dropped at the small movement from the original focusing spot then slower when the distance is larger than 100 μm. This intensity decrease actually indicates the speckle decorrelation, which can be calculated by the formula for memory effect^32,33^ as below:

2 $$C(\theta ,L)=\frac{{k}_{0}\theta L}{\sinh ({k}_{0}\theta L)};\,\sinh \,(x)=\frac{{e}^{x}-{e}^{-x}}{2}$$

where *C* is the speckle correlation coefficient (in this case, it is proportional to our spot intensity); ${k}_{0}=2\pi /\lambda$ is the wave number; $\theta$ is the angle difference from the original optimized spot, which is α_x_ or α_y_ when we scan along x or y axis; and *L* is an effective thickness of the scattering medium. From here, we can also estimate the effective thickness of our scattering medium as 96 μm.

**Figure 4:**
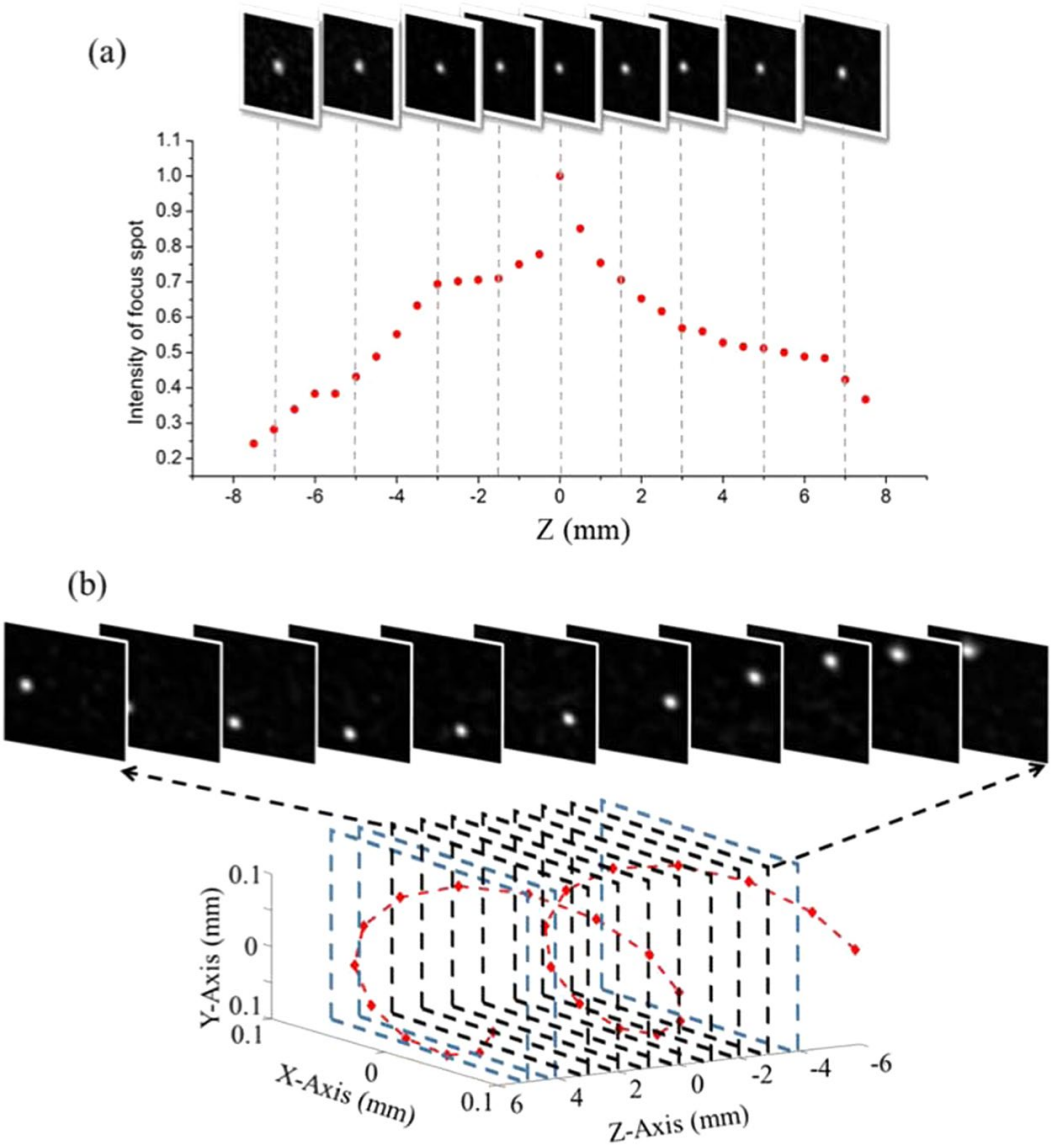
(**a**) Intensity of the moving spot along Z-axis. (**b**) Moving the focus spot in 3D space to draw a helix curve within memory effect volume. All the small insets in both (**a**) and (**b**) are the 2D images taken by a camera at corresponding Z planes.

In order to move the focus spot in Z direction, we add a quadratic phase modulation on the original phase pattern. For a small movement of $\Delta Z$, the added quadratic phase pattern is

3 $$Phase\,(\Delta Z)=k\times (\sqrt{({n}_{x}^{2}+{n}_{y}^{2})\times {p}^{2}+{(d+\Delta Z)}^{2}}-\sqrt{({n}_{x}^{2}+{n}_{y}^{2})\times {p}^{2}+{d}^{2}}-\Delta Z)$$

where $k=2\pi /\lambda$ is the wave-number, *p* is the pixel size of SLM. In case of $\Delta Z$ is much smaller than *d*, the Eq. (3) could be approximately derived to quadratic phase $[k{\rho }^{2}\Delta Z/2{d}^{2}]$^34^, where ${\rho }^{2}=({n}_{x}^{2}+{n}_{y}^{2})\times {p}^{2}$. The quality of the moving focus spot in Z-axis is demonstrated in Fig. 4a. We are able to move the spot in front and back of the original spot which is the brightest and optimized by GA algorithm. 2D images of the spots are captured by moving the camera on a precise moving stage to the expected position of each spots. Similar to X-Y plane, the intensity distribution in Z-axis is not symmetric either. This observation can be explained by the difference between moving direction of camera and the axis of moving focus spots and other imperfections in our optical setup. The efficient distance of moving spot here is about 3.3 mm forward and backward, which is 54 times larger than the longitudinal memory effect in X-Y plane $(\Delta {x}_{ME})$ or $(\Delta {y}_{ME})$. Such large memory effect in Z axis $(\Delta {z}_{ME})$ is expected in our focusing system with low numerical aperture (NA)^34^: $\Delta {z}_{ME}\approx 2\frac{d}{D}\,\Delta {x}_{ME}=\frac{1}{NA}\,\Delta {x}_{ME}$, where d (3.91 cm) is distance from scattering medium to observation camera and D (1.5 mm) is the diameter of beam shined on surface of the scattering medium.

With the ability to move the focus spot in X-Y-Z direction, our approach is capable of drawing any contours in 3D space with only single optimization process. One example is showed in Fig. 4b, where every spot are designed to draw a helix curve displayed as red dash line. We display one spot at a time within a 3D volume space of memory effect around the original spot. Again, the camera is moving along the Z axis to capture the 2D image at different planes as displayed in the insets.

### Display multiple focus spots simultaneously

**Figure 5:**
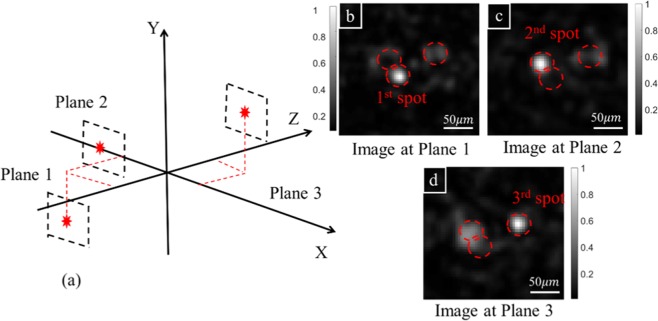
Creating 3 focusing spots simultaneously at different positions in 3D space. (**a**) Positions of 3 spots in Cartesian coordinate. (**b**–**d**) Image at the plane of the 1^st^, 2^nd^, and 3^rd^ focus spot, respectively.

In addition to moving the focus spot in 3D space sequentially, it is possible to create multiple focus spots simultaneously by using just a single phase pattern on SLM, which can be calculated by an expression below.

4 $$P={\rm{\arg }}\,[\mathop{\sum }\limits_{j=1}^{m}\,(\exp \,(i{P}_{j}))]$$

where *P* is the phase pattern to be used for creating simultaneously multiple points; *P*_*j*_ is the phase pattern for creating *j*-th single point, which is calculated from the optimized phase pattern for initial focus point. We create 3 spots on 3 different Z planes simultaneously by combining three corresponding phase patterns according to Eq. (4). Figure 5 presents the experimental results where the camera captures 2D images at different planes. At the specific plane corresponding to the targeted spot, the other two spots appear as blurry dim spots, which are presenting defocusing spots (or out-of-focus spots). The result shows the ability to form multiple spots simultaneously, eventually creating 3D shapes within the memory effect region from a single optimization process. The efficiency of our strategy depends on both spot positions and the number of spots. The spot closer to the original focus spot will have a higher intensity due to memory effect. If the number of spots increases, the quality of the 3D shape will decrease rapidly due to the lower intensity of each spots (splitting of laser beam intensity into multiple spots) and increased intensity the background because of interference of multiple backgrounds (one for each individual focus spots). In addition, due to phase only modulation (Eq. 4), the generated light field and sum of all light fields for individual focusing have different amplitudes, which reduce the efficiency. One can do better with complicated setup for both phase and amplitude modulation. Nevertheless, the intensity of three spots in Fig. 5 are almost similar with 22.1% of original focus spot’s intensity.

### Increase focus spot intensity with reduced optimization time

**Figure 6:**
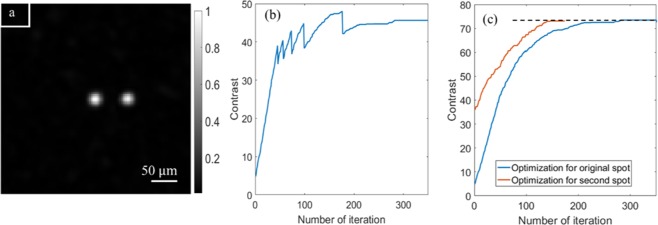
(**a**) The original focusing spot and the second focusing spot. (**b**) Optimization process to enhance the PBR of original focusing spot with background. There are 3 abrupt drops of PBR corresponding to 3 changes of exposure time due to camera saturation. (**c**) Optimization process comparison between the original focusing spot and the second focusing spot.

With a limited memory effect, we need a quick solution to increase intensity for the spots at far distance from the initial optimization point. Here, we utilize the GA to optimize the intensity again for one of the dim spots, however, with a much better initial state. All the phase patterns (i.e. the population in GA^3,24^) used for optimization of the original spot are added with a similar phase pattern to move the target to the new position within the memory effect volume. The optimization process by GA starting with the new group of phase patterns immediately achieves the dim bright spot, therefore, we expect an improvement of the optimization speed. Figure 6a shows no difference between the original focusing spot at the center and the second focusing spot. Figure 6b presents the optimization process for the original focus spot, which shows the increase of PBR after each iteration. We can also notice 5 abruptly dropping points for PBR after 45, 56, 73, 98 and 175 iterations, at which our algorithm automatically reduces the exposure time (80%) due to saturation of camera (255 counts). Lower exposure time makes the PBR dropped abruptly because of reduced signal on the background noise. It is worth to note that for our 8-bit camera (no cooling), we need to start with a reasonable exposure time to gain good signal for optimization, the intensity of focus spot increases and saturates the camera multiple times during the optimization process. Just to visualize the progress of optimization, we linearly shift the PBR up at each drops to get a monotonically increasing curve, presented as the blue curve in Fig. 6c. Similar procedure is applied to the optimization for the second spot, which starts with PBR of 35 (orange curve in Fig. 6c). There is no difference on the focusing quality; however, the optimization time is reduced by 50% for the second optimization, which inherits the set of phase patterns from the first optimization. The second focus spot can now become a center of another memory effect volume. The process can be repeated for optimizing multiple focusing spots, thus, expanding the effective scanning area for our focusing spot beyond the memory effect region.

## Conclusion

We have demonstrated an efficient approach to move the focus spot to different positions in 3D space behind the strongly scattering media. No mechanical movement is involved in our process. All the control is handled digitally with phase patterns on SLM, which is relayed on scattering medium surface through 4F optical system. More importantly, our approach relying on optical memory effect eliminates the need for multiple time-consuming optimization processes. Starting with only one original focusing spot by GA, we can move the spot around in 3D volume which is limited by memory effect. By inheriting the phase patterns from the initial optimization, we can optimize other focusing spots again at reduced time, thus, expanding the scanning region far beyond the memory effect. Finally, by combining the phase patterns for individual focusing spots on SLM, we demonstrate the multiple focus spots simultaneously. Our proposed approach could largely reduce the time for moving the focusing spot or optimizing the second spot, however, the speed is still largely limited by updating rate of the SLM. With a new solution for faster SLM such as piezo technology^35–38^, our demonstration could potentially make various biological applications possible where the focusing time can be done very fast.

## References

[1] Liu, Y. *et al*. Optical focusin0067 deep inside dynamic scattering media with near-infrared time-reversed ultrasonically encoded (TRUE) light. *Nature Communications* **6**, 5904, 10.1038/ncomms6904. 10.1038/ncomms6904 PMC4477952 25556918

[2] Miller LM, Dumas P (2006). Chemical imaging of biological tissue with synchrotron infrared light. Biochimica et Biophysica Acta (BBA) - Biomembranes.

[3] Vellekoop IM (2015). Feedback-based wavefront shaping. Opt. Express.

[4] Yoon J, Lee K, Park J, Park Y (2015). Measuring optical transmission matrices by wavefront shaping. Opt. Express.

[5] Choi Y (2011). Overcoming the Diffraction Limit Using Multiple Light Scattering in a Highly Disordered Medium. Physical Review Letters.

[6] Kim M, Choi W, Choi Y, Yoon C, Choi W (2015). Transmission matrix of a scattering medium and its applications in biophotonics. Opt. Express.

[7] Park C (2014). Full-Field Subwavelength Imaging Using a Scattering Superlens. Physical Review Letters.

[8] Park Jung-Hoon, Park Chunghyun, Yu HyeonSeung, Park Jimin, Han Seungyong, Shin Jonghwa, Ko Seung Hwan, Nam Ki Tae, Cho Yong-Hoon, Park YongKeun (2013). Subwavelength light focusing using random nanoparticles. Nature Photonics.

[9] Popoff, S., Lerosey, G., Fink, M., Boccara, A. C. & Gigan, S. Image transmission through an opaque material. *Nature Communications* **1**, 81, 10.1038/ncomms1078. 10.1038/ncomms1078 20865799

[10] Popoff SM (2010). Measuring the Transmission Matrix in Optics: An Approach to the Study and Control of Light Propagation in Disordered Media. Physical Review Letters.

[11] Zhao Q (2018). 3D focusing through highly scattering media using PSF modulation. Applied Physics Letters.

[12] Boniface A, Mounaix M, Blochet B, Piestun R, Gigan S (2017). Transmission-matrix-based point-spread-function engineering through a complex medium. Optica.

[13] Zhang J, Pégard N, Zhong J, Adesnik H, Waller L (2017). 3D computer-generated holography by non-convex optimization. Optica.

[14] Cui M, Yang C (2010). Implementation of a digital optical phase conjugation system and its application to study the robustness of turbidity suppression by phase conjugation. Opt. Express.

[15] Hsieh C-L, Pu Y, Grange R, Laporte G, Psaltis D (2010). Imaging through turbid layers by scanning the phase conjugated second harmonic radiation from a nanoparticle. Opt. Express.

[16] Hsieh C-L, Pu Y, Grange R, Psaltis D (2010). Digital phase conjugation of second harmonic radiation emitted by nanoparticles in turbid media. Opt. Express.

[17] Katz Ori, Heidmann Pierre, Fink Mathias, Gigan Sylvain (2014). Non-invasive single-shot imaging through scattering layers and around corners via speckle correlations. Nature Photonics.

[18] Yang X, Hsieh C-L, Pu Y, Psaltis D (2012). Three-dimensional scanning microscopy through thin turbid media. Opt. Express.

[19] Yaqoob Zahid, Psaltis Demetri, Feld Michael S., Yang Changhuei (2008). Optical phase conjugation for turbidity suppression in biological samples. Nature Photonics.

[20] Shibukawa A, Okamoto A, Goto Y, Honma S, Tomita A (2014). Digital phase conjugate mirror by parallel arrangement of two phase-only spatial light modulators. Opt. Express.

[21] Liu Y, Ma C, Shen Y, Shi J, Wang LV (2017). Focusing light inside dynamic scattering media with millisecond digital optical phase conjugation. Optica.

[22] Yang J, Shen Y, Liu Y, Hemphill AS, Wang LV (2017). Focusing light through scattering media by polarization modulation based generalized digital optical phase conjugation. Applied Physics Letters.

[23] Yang J, Li J, He S, Wang LV (2019). Angular-spectrum modeling of focusing light inside scattering media by optical phase conjugation. Optica.

[24] Conkey DB, Brown AN, Caravaca-Aguirre AM, Piestun R (2012). Genetic algorithm optimization for focusing through turbid media in noisy environments. Opt. Express.

[25] Vellekoop IM, Aegerter CM (2010). Scattered light fluorescence microscopy: imaging through turbid layers. Opt. Lett..

[26] Yu Hyeonseung, Lee KyeoReh, Park Jongchan, Park YongKeun (2017). Ultrahigh-definition dynamic 3D holographic display by active control of volume speckle fields. Nature Photonics.

[27] Freund I, Rosenbluh M, Feng S (1988). Memory Effects in Propagation of Optical Waves through Disordered Media. Physical Review Letters.

[28] Osnabrugge G, Horstmeyer R, Papadopoulos IN, Judkewitz B, Vellekoop IM (2017). Generalized optical memory effect. Optica.

[29] Judkewitz Benjamin, Horstmeyer Roarke, Vellekoop Ivo M., Papadopoulos Ioannis N., Yang Changhuei (2015). Translation correlations in anisotropically scattering media. Nature Physics.

[30] Tran, V., Sahoo, S. K., Tang, D. & Dang, C. In *SPIE BiOS*. 9 (SPIE) (2018).

[31] Xu J, Ruan H, Liu Y, Zhou H, Yang C (2017). Focusing light through scattering media by transmission matrix inversion. Opt. Express.

[32] Tang D, Sahoo SK, Tran V, Dang C (2018). Single-shot large field of view imaging with scattering media by spatial demultiplexing. Appl. Opt..

[33] Sahoo SK, Tang D, Dang C (2017). Single-shot multispectral imaging with a monochromatic camera. Optica.

[34] Katz Ori, Small Eran, Silberberg Yaron (2012). Looking around corners and through thin turbid layers in real time with scattered incoherent light. Nature Photonics.

[35] Callamaras N, Parker I (1999). Construction of a confocal microscope for real-time x-y and x-z imaging. Cell Calcium.

[36] Weiner AM (2000). Femtosecond pulse shaping using spatial light modulators. Review of Scientific Instruments.

[37] Dal Maschio M, De Stasi AM, Benfenati F, Fellin T (2011). Three-dimensional *in vivo* scanning microscopy with inertia-free focus control. Opt. Lett..

[38] Stockbridge C (2012). Focusing through dynamic scattering media. Opt. Express.

